# Precipitation of Phosphate Minerals by Microorganisms Isolated from a Fixed-Biofilm Reactor Used for the Treatment of Domestic Wastewater

**DOI:** 10.3390/ijerph110403689

**Published:** 2014-04-02

**Authors:** Almudena Rivadeneyra, Alejandro Gonzalez-Martinez, Jesus Gonzalez-Lopez, Daniel Martin-Ramos, Maria Victoria Martinez-Toledo, Maria Angustias Rivadeneyra

**Affiliations:** 1 Department of Electronic and Computer Technology, Higher Technical School of Computer Engineering and Telecommunications , University of Granada, University Campus of Almanjayar, Granada 18071, Spain; E-Mail: arivadeneyra@ugr.es; 2 Department of Civil Engineering, Advanced Technical School of Civil Engineers, University of Granada, Fuentenueva Campus, Granada 18071, Spain; 3 Departament of Microbiology, Faculty of Pharmacy, University of Granada, University Campus of Cartuja, Granada 18071, Spain; E-Mails: jgl@ugr.es (J.G.-L.); mvmt@ugr.es (M.V.M.-.T.); mrivaden@ugr.es (M.A.R.); 4 Department of Mineralogy and Petrology, Faculty of Science, University of Granada, Fuentenueva Campus , Granada 18071, Spain; E-Mail: jdmartin@ugr.es

**Keywords:** struvite, spherulites, bobierrite, baricite, urban wastewater, phosphate precipitation, submerged fixed-film bioreactor

## Abstract

The ability of bacteria isolated from a fixed-film bioreactor to precipitate phosphate crystals for the treatment of domestic wastewater in both artificial and natural media was studied. When this was demonstrated in artificial solid media for crystal formation, precipitation took place rapidly, and crystal formation began 3 days after inoculation. The percentage of phosphate-forming bacteria was slightly higher than 75%. Twelve major colonies with phosphate precipitation capacity were the dominant heterotrophic platable bacteria growing aerobically in artificial media. According to their taxonomic affiliations (based on partial sequencing of the 16S rRNA), the 12 strains belonged to the following genera of Gram-negative bacteria: *Rhodobacter*, *Pseudoxanthobacter*, *Escherichia*, *Alcaligenes*, *Roseobacter*, *Ochrobactrum*, *Agromyce*, *Sphingomonas* and *Paracoccus*. The phylogenetic tree shows that most of the identified populations were evolutionarily related to the Alphaproteobacteria (91.66% of sequences). The minerals formed were studied by X-ray diffraction, scanning electron microscopy (SEM), and energy dispersive X-ray microanalysis (EDX). All of these strains formed phosphate crystals and precipitated struvite (MgNH_4_PO_4_·6H_2_O), bobierrite [Mg_3_(PO_4_)_2_·8H_2_O] and baricite [(MgFe)_3_(PO_4_)_2_·8H_2_O]. The results obtained in this study show that struvite and spherulite crystals did not show any cell marks. Moreover, phosphate precipitation was observed in the bacterial mass but also near the colonies. Our results suggest that the microbial population contributed to phosphate precipitation by changing the media as a consequence of their metabolic activity. Moreover, the results of this research suggest that bacteria play an active role in the mineral precipitation of soluble phosphate from urban wastewater in submerged fixed-film bioreactors.

## 1. Introduction

It is widely accepted that microorganisms contribute to the bioprecipitation of a wide variety of minerals, including carbonates, phosphates, sulphides, oxides, and silicates [1]. Different mechanisms have been proposed for mineral precipitation by microorganisms in natural and artificial habitats [1,2,3,4,5]. Nevertheless, in many cases, the exact role of microbes in this biological process is not known, and there is some controversy as to whether they play a passive or active role, and whether they can directly influence the mineralogy of precipitates [6]. In this context, different microbial species have previously been reported and assumed to be associated with biomineral precipitation in diverse environments including bioreactor systems for industrial or urban wastewater treatments [7,8]. Species-specific mineral precipitation has been suggested by several authors [9,10,11], though the exact mechanisms of precipitation and the way in which this process works within the microbial ecology of the precipitating organism has still not been resolved.

Struvite (MgNH_4_PO_4_·6H_2_O) is not a very abundant mineral in Nature; it has only been found in association with organic matter decomposition in locations such as barns, sheds, cemetery soils, guano deposits, manures, sediments rich in organic remains, and in kidney stones [9,10,11,12] Struvite is also frequently produced in wastewater treatment plants [13,14]. Several authors have connected the precipitation of struvite in different natural habitats with microbial activity and have reported struvite production by different bacterial strains [3,15,16].

Struvite in wastewater treatment plants was identified as early as 1939 and its formation is often associated with anaerobic and postdigestion processes [13]. Problems with struvite formation date back to the 1960s when it was noticed at the Hyperion treatment plant, Los Angeles (CA, USA), where the digested sludge pipeline diameter decreased from 12 to 6 cm [17]. Similar instances of pipe blockages have been reported elsewhere [18,19]. In this context, there is growing interest in using specific reactors for struvite precipitation because of the wide range of operational problems caused by struvite accumulation in wastewater treatment plants [14].

Submerged fixed-film bioreactor technologies are wastewater treatment systems in which the organic matter in wastewater provides an energy source for the production of new cells for a mixed population of microbes in an aquatic aerobic or anaerobic environment. This paper describes a study of phosphate precipitation by heterotrophic bacteria isolated from a submerged fixed-film bioreactor in culture media consisting of urban wastewater and artificial culture media (conventional media for phosphate precipitation). The objective of this research was to discover which culture conditions influenced biomineral formation by the isolated bacteria and confirm the active role played by these bacteria in the mineral precipitation of soluble phosphate from urban wastewater in submerged fixed-film bioreactors.

## 2. Materials and Methods

### 2.1. Bench-Scale Experimental Plant and Operating Conditions

**Figure 1:**
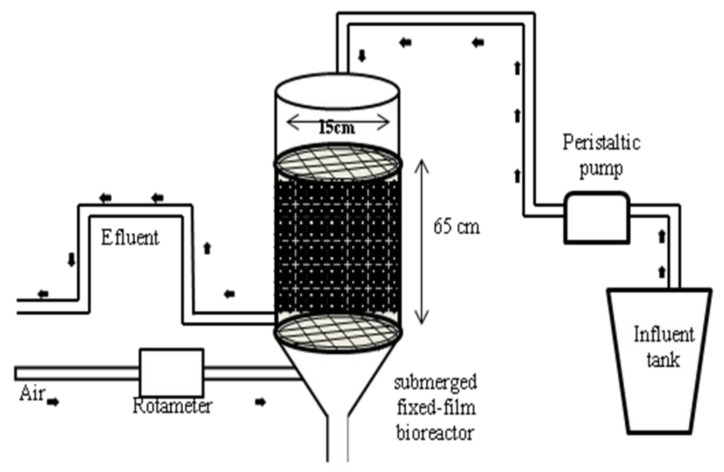
Diagram of submerged fixed-film bioreactor used for urban wastewater treatment constructed at bench scale and used in this study.

The bench-scale plant used for this study (Figure 1) was based on a design that had been used for urban wastewater treatment in previous studies [20,21]. It consisted of a methacrylate cylindrical column with a bed size of 65 cm in height and a 15 cm diameter. A porous plastic carrier, Bioflow 9 (RVT Company, Knoxville, TN, USA), with a surface of 800 m^2^/m^3^ and a bulk density of 145 kg/m^3^ was used as carrier material. Air was supplied by a diffuser placed on the bottom of the reactor. It was fed with influent urban wastewater (72 L/day) coming from the primary settling tank of a local municipal wastewater treatment plant (WWTP, “EDAR SUR”, EMASAGRA S.A., Granada, Spain).

Bioreactor cleaning cycles were performed every 15 days in order to avoid filter clogging. The experiments were carried out with an inflow rate of 50 mL/min, hydraulic retention time (3.8 h), and a constant temperature of 20 °C, according to a previous study [21]. These working conditions had a total duration of 45 days, divided into three cycles of 15 days due to clogging of the biofilter.

### 2.2. Physico-Chemical Analysis

Influent and effluent wastewater samples from the bioreactor were obtained every 24 h for physico-chemical studies. The biological oxygen demand at 5 days (BOD5), chemical oxygen demand (COD), total suspended solids (TSS), and volatile suspended solids (VSS) were determined according to Standard Methods for the Examination of Waste and Wastewater [22]. The pH was determined with a Crison pH 25 pH-meter (Crison Instruments S.A., Barcelona, Spain). Physico-chemical data of the bioreactor under experimental conditions were analysed using the software package Statgraphics 5.0 (STSC, Rockville, MD, USA) to analyse the variance (analysis of variance [ANOVA]). A significance level of 95% (*p <* 0.05) was employed.

### 2.3. Biofilm Recovery from the Carrier Material

Biofilm samples from the carrier material were recovered from the three consecutive cycles of operation (every 15 days) by the following method: 10–100 g of carrier material with biofilm adhered was taken and placed in flasks with 50 mL of sterile saline (0.9% NaCl). In order to detach the biofilm from the carrier and disperse cells, the suspensions were sonicated in a bath sonicator (Selecta, Barcelona, Spain) at room temperature for 2 min at 40 kHz (0.05 W/mL) and then placed in an orbital shaker at 155 rpm for 1 h. The process was performed twice. The suspensions of biofilm material were then used for the isolation of heterotrophic bacteria.

### 2.4. Microorganisms and Culture Media

Aliquots (1 mL) of the biofilm samples were serially diluted and spread on wastewater solid media (WWM) and two artificial solid media (SS1 and SS2). WWM medium was composed of urban wastewater. Specific media for phosphate precipitation (SS1 and SS2) were composed of 10.0 g/L yeast extract, 5.0 g/L protease peptone, and 1.0 g/L glucose. SS1 medium was supplemented with 4.0 g/L MgSO_4_·7H_2_O and 2.5 g/L K_2_HPO_4_ [23], while SS2 medium was supplemented with 8.0 g/L of magnesium acetate [24]. To obtain solid media, 18 g/L Bacto-Agar was added, and the pH was adjusted to 7.2 with 0.1 M KOH. The culture media were autoclaved at 112 °C for 20 min.

Wastewater samples for the preparation of the media were collected from the wastewater treatment plant of the city of Granada. This wastewater was taken from the primary settling tank of the treatment plant and was used as influent in the bench-scale experimental plant (submerged fixed-biofilm reactor) located in the Water Research Institute (University of Granada). The average content of total phosphate and ammonia in the wastewater was 12 mg/L and 65 mg/L, respectively.

All the inoculated solid media, after 72 h of aerobic cultivation at 25 °C, showed single, morphologically well-formed colonies. The plates were checked periodically for the presence or absence of phosphate crystals using optical microscopy. Isolated representatives of the dominant colony morphologies with phosphate-forming capacity in SS1 and SS2 (12 major colony types) were selected and purified, by restreaking them twice.

The experiments in liquid media were performed using inoculated biofilm suspensions on SSL1 medium and SSL2 medium. The main goal of these experiments was to demonstrate phosphate precipitation in liquid media. Erlenmeyer flasks of 1 L of capacity containing 250 mL of SSL1 and SSL2 liquid media were inoculated with 10 mL of biofilm suspensions obtained as described above. The cultures were then aerobically incubated at 25 °C for 30 days. The evolution of pH was monitored throughout the experiment using the Crison Basic 20 pH meter. Precipitates were removed by centrifugation of the media and the sediments were re-suspended and washed in distilled water to free them of impurities. The washed carbonate crystals were finally air-dried at 37 °C.

A control consisting of uninoculated culture media and media inoculated with autoclaved cells (dead cells) were included in all experiments. All the experiments were performed two times.

### 2.5. Study of Mineral Formation

For the study of phosphate precipitation, all the selected strains (major colony types) were surface-inoculated onto SS1 and SS2 solid media and incubated aerobically at 25 °C. Thirty days after inoculation, struvite crystals were extracted from the solid agar media by means of a small spatula and washed with distilled water to free them of impurities. Spherulitic crystals were removed from the solid media by cutting out pieces, which were placed in boiling water to dissolve the agar. The supernatants and the sediments were resuspended and washed in distilled water to free them of impurities.

Precipitates obtained in liquid media were removed by centrifugation of the media and the sediments were re-suspended and washed in distilled water to free them of impurities. All the washed phosphate crystals were finally air-dried at 37 °C. Using this methodology, the morphology of crystals was not altered, as observed by optical microscopy both before and after their recovery.

### 2.6. Identification of Bacterial Strains

All the strains (with phosphate precipitation capacity) were taxonomically classified by analysing the partial sequence of the gene encoding 16S rRNA. Primers fD1 and rD1 [25] were synthesised by Sigma Genosis (San Louis, MO, USA) and used to amplify nearly the full length of the 16S rRNA gene. Fresh cultured colonies of each strain were lysed by the addition of 20 µL of a mixture of NaOH (0.05 M)-SDS (0.25 %, w/v), which was then boiled for 15 min. The lysates were adjusted to 200 µL with sterile water and centrifuged at 2500 g for 5 min in a table-top centrifuge. The cleared lysates (4 µL) were used as a template for amplification. PCR was carried out by adding the following to the lysates: 1 × PCR Gold buffer (Applied Biosystems, Darmstadt, Germany); 1.5 mM MgCl_2_ (Applied Biosystems); 200 µM dNTPs (Roche Molecular Biochemicals, Mannheim, Germany); 20 pmol of each primer; and 1 U of Ampli-Tag Gold polymerase (Applied Biosystems). The final volume of the reaction tubes was adjusted to 50 µL. Reactions were run in a Perkin Elmer GeneAmp PCR system 2400 (Perkin Elmer, Norwalk, CT, USA). The temperature profile was as previously described by Vinuesa *et al.* [26], except for extension of the initial denaturation step to 7 min, as required, using the Quiaex II kit (Qiagen, Düsseldorf, Germany). The nucleotide sequence of the purified bands was determined by the dideoxy chain terminator method, using the ABI-PRISM Big Dye Terminator Cycle Sequencing Ready Reaction kit (Perkin Elmer) and automated sequencer ABI-PRISM 3100 Avant Genetic Analyzer (Applied Biosystems). The sequenced fragment analysed corresponded to the first 650 bp of the 16S rRNA gene, comprising hypervariable regions V1, V2, and V3 [27]. DNA sequences were analysed using the biocomputing tools provided on-line by the European Bioinformatics Institute (http://www.ebi.ac.uk/ services/dna-rna). The BLASTn [28] program was used for preliminary sequence similarity analysis, and the ClustalX v.1.8 software [29] was used for sequence alignment. Phylogenetic and molecular evolutionary analysis was conducted using MEGA version 4 [30]. A *p*-distance-based evolutionary tree was inferred using the Neighbour-Joining algorithm. The bootstrap test was conducted to infer the reliability of branch order, with a round of 1000 reassembling. Bootstrap values below 50% are not shown in the tree.

### 2.7. X-Ray Diffraction Study

The minerals obtained from solid and liquid media were examined by powder X-ray diffraction (PXRD) using a Philips PW 1710/00 diffractometer (Royal Philips Electronics, Amsterdam, The Netherlands) with a graphite monochromator automatic slit, CuKα radiation, and an on-line connection with a microcomputer. Data were collected for a 0.4 s integration time in 0.02° 2 steps at 40 kV and 40 mA in a 2 interval between 3–80°. Data were processed using the XPowder program for qualitative and quantitative determination of the mineral composition.

### 2.8. Scanning Electron Microscopy Study

Secondary electron micrographs of phosphate precipitates were made with gold-coated samples using a Zeiss DMS SEM (LEO Electron Microscopy, Oberkochem, Germany), operated at an acceleration voltage of 20 kV to examine the micromorphology of the crystals. Selected samples were coated with carbon for energy dispersive X-ray (EDX) microanalysis. High-resolution secondary electron images were prepared with a LEO 1525 field-emission scanning electron microscope (FESEM) under 2–3 kV on carbon-coated samples.

### 2.9. Statistical Analysis

Physico-chemical data of the bioreactor under experimental conditions were analysed using the software package Statgraphics 5.0 to analyse the variance (ANOVA). A significance level of 95% (*p <* 0.05) was employed.

## 3. Results and Discussion

In recent years, there have been important advances in biological treatment technologies such as MBR systems and submerged fixed-bed biofilm reactor systems for the removal of nutrients, such as organic matter, phosphate, and nitrogen [20]. Thus, in the last 25 years, intensive research in the field of biological wastewater treatment (including MBR and submerged biofilter) has shown that fixed-biofilm systems are often more efficient for water purification than conventional suspended activated sludge [31].

Table 1 shows the average values of the COD, BOD5, TSS, VSS, and pH detected in the bioreactor (influent and effluent) during the study. As previously reported [32], submerged filter systems have been shown to be highly efficient at removing COD and BOD5 from urban wastewater. The system used in our study was no exception, presenting an average 92% reduction of COD concentration in the effluent with respect to the influent. However, it must be remarked that the bioreactor was cleaned every 15 days (three times during one experiment), and consequently the elimination of organic matter by filtration must also be considered in the submerged bioreactor. The generation of an active biofilm from the microbiota normally present in urban wastewater takes place rapidly because of the high microbial population and nutrient concentration in these environments. In our case, a stable biofilm was observed in the bioreactor after 24–48 h under our experimental conditions. Biofilm formation in the submerged filter was confirmed by optical microscopy.

**Table 1:** Operational conditions in the submerged fixed-film bioreactor at bench scale during the experiments.

Parameter	Influent ^1^	Effluent ^2^	Reduction %	LSD
BOD5 (mg O_2_/L)	425.65 ± 97.83	17.24 ± 6.51	95.95	23.87
COD (mg O_2_/L)	980.26 ± 233.05	78.47± 28.64	92.00	48.12
pH	7.55 ± 0.21	7.46 ± 0.26	-	0.12
TSS (mg/L)	622.43 ± 398.95	19.48 ± 7.61	96.87	4.05
VSS (mg/L)	520.48 ± 153.24	13.10 ± 6.14	97.48	3.86

Average values marked with the same letter are not significantly different, according to the least significant difference (LSD) test (*p <* 0.05). ^1^ Urban wastewater; ^2^ Urban wastewater after treatment. The working conditions had a duration of 45 days.

**Figure 2:**
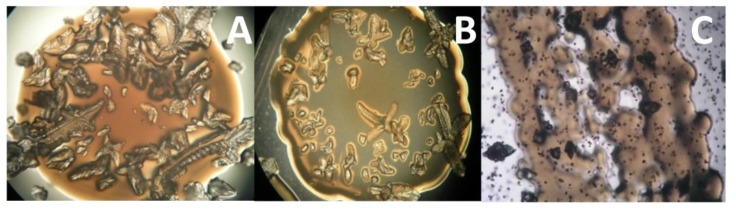
Growth of colonies with precipitates of phosphate in SS1 and SS2 solid media. (**A**): Struvite-forming colony in SS1 medium; (**B**): Struvite-forming colony in SS2 medium; (**C**): Struvite and spherulites biomass in SS1 medium.

The formation of phosphate crystals in solid media was only detected in artificial media (SS1 and SS2). However, no formation was observed in WWM, uninoculated control media, or media inoculated with a high concentration of dead bacteria without metabolic activity. The number of heterotrophic bacteria (colony forming units, CFU) per mL of biofilm in WWM, SS1, and SS2 media was in the range of 5.5 × 10^5^, 3.3 × 10^6^, and 2.8 × 10^6^, respectively. The percentage of phosphate-forming bacteria in artificial media SS1 and SS2 was 75.1% and 77.7%, respectively. Crystal formation took place rapidly, beginning at 3 days after inoculation. After 10 days, the crystals had significantly increased in quantity and were of a large size. Figure 2 shows colonies with phosphate precipitates.

Twelve major colony types that produced large amounts of phosphate crystals were selected for taxonomical identification. The taxonomic affiliations of the selected strains, based on partial sequencing of the 16S rRNA gene (V1 to V3 hypervariable regions, ca. 650 nt) are shown in Table 2. The strains belonged to eight different genera of Gram-negative bacteria. Sequence comparison with databases demonstrated the affiliation of strain 1 to *Rhodobacter* sp. (100% identity), strain 2 to *Pseudoxanthobacter* sp. (98% identity), strain 3 to *Escherichia coli* (100% identity), strain 4 to *Alcaligenes* sp. (100% identity), strain 5 to *Roseobacter* sp. (98 identity), strain 6 to *Agrobacterium* sp. (100% identity), strain 7 to *Ochrobactrum anthropi* (100% identity), strain 8 to *Sphingomonas* sp. (98% identity), strain 9 to *Paracoccus* sp. (100% identity), strain 10 to *Agrobacterium tumefaciens* (100% identity), strain 11 to *Sphingomonas adhesiva* (100% identity), and strain 12 to *Rhodobacter* sp. (96% identity).

**Table 2:** Taxonomic analysis of struvite-forming bacteria from the data bank NCBI.

Strain	Identities	% Similarity	Name sequence reference
1	191	100	HM124369.1 *Rhodobacte*r sp.
2	198	98	FJ587218.1 *Pseudoxanthobacter* sp.
3	190	100	HM629504.1 *Escherichia coli* strain BAB-286
4	174	100	EF195167.1 *Alcaligenes* sp.
5	179	98	AY576768.1 *Roseobacter* sp.
6	192	100	EU295451.1 *Agrobacterium* sp.
7	184	100	GU415542.1 *Ochrobactrum anthropi* AW034
8	175	98	AB033946.1 *Sphingomonas* sp.
9	107	100	DQ465245.1 *Paracoccus* sp.
10	198	100	JX293316.1 *Agrobacterium tumefaciens*
11	192	100	AB680766.1 *Sphingomonas adhaesive*
12	191	100	HM124369.1 *Rhodobacter* sp.

**Figure 3:**
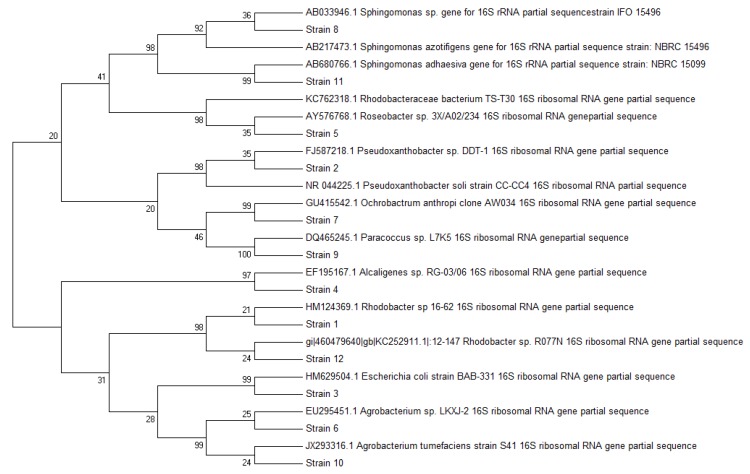
Neighbor-joining phylogenetic tree showing the position of 12 bacterial sequences and the most similar sequences retrieved from the EMB database, based on ca. 200 nt length of sequences.

The phylogenetic tree (Figure 3) shows that most of the identified populations were evolutionarily related to the Alphaproteobacteria (91.66% of sequences). One sequence exclusively represented populations phylogenetically close to Gammaproteobacteria. According to our results, Alphaproteobacteria were identified as the dominant group of bacteria involved in phosphate precipitation in the submerged fixed-bed biofilm reactor system. Alphaproteobacteria are widespread components of bacterial community in biofilm systems [33], where members of the phylogenetic group are responsible for the processes of degradation of organic matter during wastewater treatment, and they display an ability to become part of heterogeneous biofilms formed under such conditions.

Numerous laboratory studies in natural environments have demonstrated the microbial precipitation of struvite [3,15,25,34,35]. Nevertheless, no research has been published on the bioprecipitation of these minerals associated with submerged fixed-film bioreactors. Our study did not detect the formation of minerals such as struvite when bacterial colonies were grown in a natural culture media containing urban wastewater as a source of nutrients (WWM medium). In the case of the WWM medium, all the bacterial strains assayed grew very well and formed colonies in 48–72 h, though no crystal formation was detected after the 30-day incubation period. In contrast, when bacterial strains were grown in artificial culture media (solid or liquid media), phosphate precipitation occurred after an incubation period of 3 days. Our data suggest that the nutrient concentration or nutrient composition in the urban wastewater in our experiment was not sufficient to produce the precipitation of phosphate under our experimental conditions. However, in artificial culture media supplemented with high concentrations of metabolisable organic matter, phosphate and magnesium, or only magnesium, the bacterial populations were able to create optimal conditions for the formation of crystals.

Our study also showed a significant number of strains (50%) with the ability to precipitate spherulites of the phosphate compounds bobierrite [Mg_3_(PO_4_)_2_·8H_2_O] and baricite [(MgFe)_3_(PO4)_2_·8H_2_O] (Table 3). These minerals are rarely found in Nature. Bobierrite has been described in association with microbial activity in some specific habitats with a very high concentration of organic matter. Thus, Rivadeneyra *et al.* [36] reported the precipitation of bobierrite by *Acinetobacter* sp. in natural environments. However, the description of bacterial strains able to precipitate phosphates as bobierrite is practically unknown. Our results show that different bacterial strains isolated from a fixed-biofilm bioreactor used for the treatment of urban wastewater had the capacity to precipitate phosphates as bobierrite and baricite when grown in SS1 and SS2 solid media, while this capacity was not detected in liquid media (SSL1 and SSL2). Moreover, the formation of both phosphates (bobierrite and baricite) took place at the same time as that of struvite crystals. These data suggest that the precipitation of bobierrite by microorganisms may be more common than expected. This is the first report on bacterial precipitation of baricite by bacteria in laboratory cultures.

The most important finding of this research study is that the bacterial population isolated from a submerged fixed-film bioreactor was able to precipitate phosphate. However, in our study precipitation capacity only occurred when the bacteria were cultivated in artificial laboratory media but not when the bacteria were cultivated in natural media derived from urban wastewater. This suggests that in real urban wastewater, the precipitation of magnesium phosphate through bacterial action can take place when the wastewater has a high concentration of nutrients, such as magnesium or phosphate and magnesium.

**Table 3:** Formation of phosphate crystals by bacterial strains isolated from submerged fixed-film bioreactor in solid and liquid media.

Strain	SS1 medium	SS2 medium	SSL1 medium	SSL2 medium
1	Struvite + spherulities	Struvite + spherulities	Struvite	Struvite
2	Struvite + spherulities	Struvite + spherulities	Struvite	Struvite
3	Struvite	Struvite	Struvite	Struvite
4	Struvite	Struvite	Struvite	Struvite
5	Struvite + spherulities	Struvite + spherulities	Struvite	Struvite
6	Struvite	Struvite	Struvite	Struvite
7	Struvite + spherulities	Struvite + spherulities	Struvite	Struvite
8	Struvite	Struvite	Struvite	Struvite
9	Struvite + spherulities	Struvite + spherulities	Struvite	Struvite
10	Struvite	Struvite	Struvite	Struvite
11	Struvite	Struvite	Struvite	Struvite
12	Struvite + spherulities	Struvite + spherulities	Struvite	Struvite

**Figure 4:**
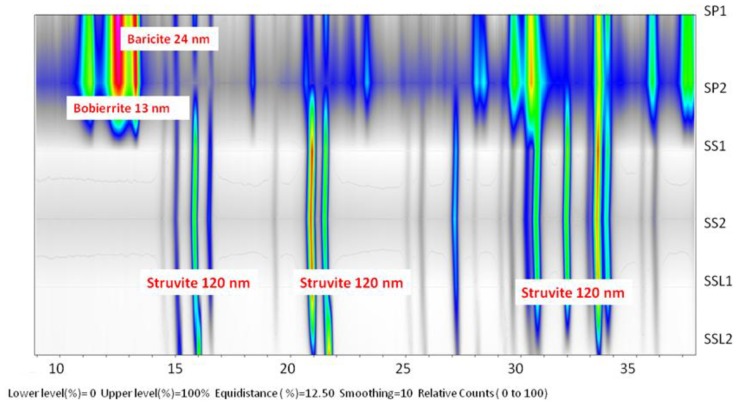
Map drawn from X-ray diffraction (XRD) patterns. *Colors* in the map indicate the changing intensity of the diffracted X-rays as a function of 2, with warmer colors for progressively higher intensities.

The results of mineralogical analysis with XRD (Figure 4, Table 3) showed that the phosphate precipitated in the SS1 medium and SS2 medium was struvite and spherulite compounds of bobierrite and baricite.

**Figure 5:**
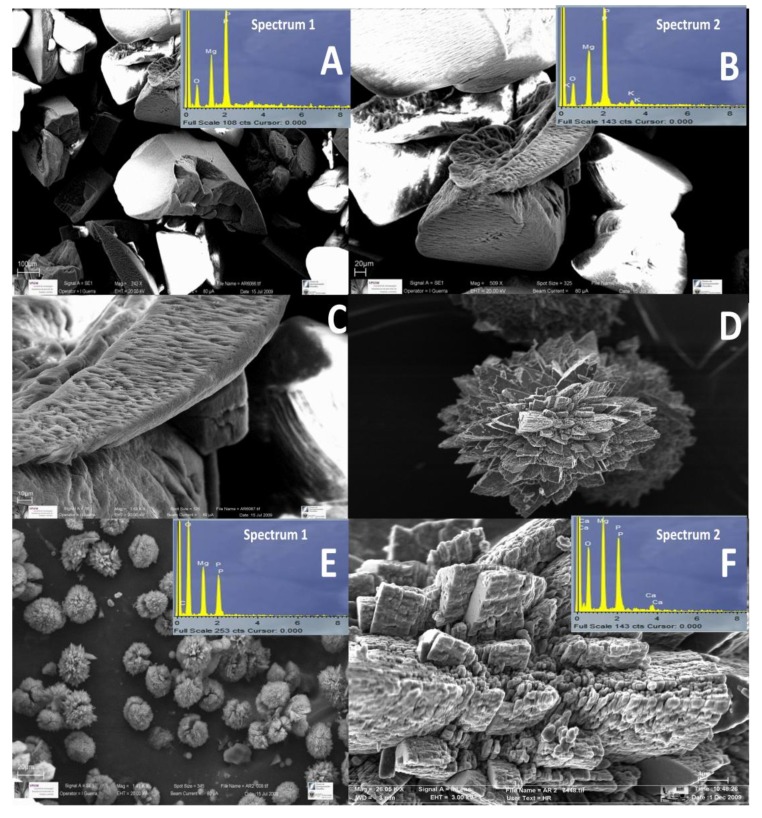
Photomicrographs of scanning electron microscope (SEM) and field emission scanning electron microscope of struvite crystals (**A**–**C**) and bobierrite and Mg_3_(PO_4_)_2_·2H_2_O spherulites (**D**–**F**) formed in solid media where certain morphological details can be seen. Energy dispersive X-ray microanalysis (EDX) spectra of struvite crystals and spherulites are included in the figure.

Struvite crystals had polyhedral shapes with a high degree of crystallinity. EDX analysis (Figure 5) confirmed the XRD results. SEM and FESEM observations were also used to study the morphological characteristics of the biominerals precipitated (Figure 5). Thus, SEM observation of struvite and spherulites did not show any cell marks. Optical microscopic observation of the plates showed that struvite and spherulites were located in the bacterial mass and also near the colonies (Figure 2).

The influence of microorganisms on mineral precipitation has been recognised for a wide variety of minerals. In fact, there have been various research studies on microbial involvement in carbonate and phosphate precipitation in natural environments [34,35,37]. Generally speaking, phosphate biomineralisation is not necessarily linked to any specific group of microorganisms, although these biological processes have been reported in many different environments. However, phosphate biomineralisation by bacteria in a submerged fixed-film bioreactor has not as yet been studied. In fact, very little is known about the biomineralisation capacity of heterotrophic bacteria in natural or artificial environments (particularly in wastewater treatment systems) and their precipitation mechanism.

Rivadeneyra *et al.* [2] proposed a mechanism for struvite precipitation in which the adsorption of Mg^2+^ and PO_4_^3−^ ions, together with the release of NH_4_^+^ ion, may be responsible for its formation. A similar mechanism may occur in our study because the culture media used contained Mg^2+^ and PO_4_^2+^, as well as peptone and yeast extract as organic matter. In this context, metabolisation of the organic matter produces NH_4_^+^ and PO_4_^3−^ ions, which are required for the precipitation of struvite minerals. In this mechanism, the metabolic activity of the bacteria is extremely important because it supplies the ions NH_4_^+^ and PO_4_^3−^ that are necessary for struvite formation. Moreover, the importance of the metabolic activity in the biomineralisation process is supported by the fact that no precipitation was observed in control experiments without bacteria or with autoclaved cells. Our results confirm this precipitation mechanism and suggest that the struvite in wastewater treatment systems could be produced by bacterial populations able to create the optimal conditions (such as enough concentrations of phosphate and NH_4_^+^) for the formation of struvite crystals.

The influence of microbial composition in the biofilm is a significant factor that affects the application of submerged fixed-film bioreactors for water treatment [38]. In this context, biofilm is very complex habitat where the microbial cells responsible for the treatment process are embedded in a polymer matrix. As previously described, the main components of the biofilm are physiologically and morphologically different bacterial cells and their composition depends on environmental conditions [20]. *Alphagammabacteria* are ubiquitous microorganisms in natural habitats and our results suggest that these microorganisms can also be identified as a component of the microbial population in the biofilms formed in submerged fixed-film bioreactors used for the treatment of urban wastewater. These bacteria lack special growth requirements. Furthermore, they can easily grow in low or rich-nutrient environments and produce exopolysaccharides (EPS) characteristics which are advantageous to the growth and formation of stable biofilms in submerged filters. Moreover, the results of this work have demonstrated the potential capacity of these bacterial populations in the mineral precipitation of soluble phosphate from urban wastewater. Future research will focus on the specific *in situ* quantification of these organisms in biofilters by fluorescent *in situ* hibridization (FISH).

Our study showed that struvite and spherulite crystals were free of mineralised cells. Optical microscopic observation of the plates containing SS1 and SS2 media showed that the majority of the struvite crystals were not only in the microbial masses but also near the colonies. These results suggest that the crystals were not formed by the aggregation of mineralised cells, and in those cases, bacterial cells contributed to this formation only by changing the media as a consequence of their metabolic activity. Thus, all the results of this study, which are obviously limited to cultivable bacteria isolated in laboratory media, verify that heterotrophic bacteria such as *Rhodobacter* sp., *Agrobacterium* sp., *Paracoccus* sp., *Roseobacter* sp., and *Sphingomonas* sp., have an important function in the formation of struvite studied *in vitro* and that consequently, this is a biomineralisation process. However, the significance of this biological process for nutrient removal in wastewater treatment technology must be studied in greater depth in future research.

## 4. Conclusions

The results of this study, which are limited to platable bacteria, suggest that bacterial activity has an importance role in the formation of phosphate crystals such as struvite and spherulites in submerged fixed-film bioreactors, and that consequently, this is a biomineralisation process. The precipitation of phosphate crystals by bacteria isolated from a submerged fixed-film bioreactor used for the treatment of urban wastewater, which contains a high concentration of soluble minerals and metabolisable organic matter, is mainly as struvite. However, for the study of biomineralisation by heterotrophic bacteria from submerged biofilter systems used for urban wastewater treatment, it is advisable to use media derived from urban wastewater supplemented with a high concentration of magnesium, since environmental conditions present in the bioreactor are not optimal for the precipitation of minerals. Moreover, the results of this research suggest that heterotrophic bacteria populations play an active role in mineral precipitation of soluble phosphate from urban wastewater.
